# Gray’s Personality Dimensions and Reasons for Voluntary Sleep Deprivation Among College Students

**DOI:** 10.3389/fpsyg.2018.02316

**Published:** 2018-11-23

**Authors:** Nina Andersz, Kamilla Bargiel-Matusiewicz

**Affiliations:** Faculty of Psychology, University of Warsaw, Warsaw, Poland

**Keywords:** BIS, BAS, sleep deprivation, sleep quality, temperamental traits, health-related quality of life

## Abstract

Tendency toward healthy and health-risk behavior is associated with external factors, and healthy lifestyle affects its quality. Activation of Behavioral Inhibition System (BIS) and Behavioral Activation System (BAS) and its association with sleeping habits provides insight into the correlates of voluntary sleep deprivation. Aim of the study was to evaluate the relationship between BIS/BAS activity and reasons for voluntary sleep deprivation among young adults and to assess benefits and costs of decreasing sleep duration. Participants were 223 college students. The instruments used were the BIS/BAS Scale, Pittsburgh Sleep Quality Index and a newly developed survey. Statistical analysis wasconducted using Mann-Whitney’s *U*-test and Spearman’s rho correlation. Increased BIS activity is positively correlated with the frequency of foregoing sleep to study (*r* = 0.19; *p* < 0.01), while activation of BAS Fun Seeking is associated with more frequent voluntary sleep deprivation due to work (*r* = 0.18; *p* < 0.05), social life (*r* = 0.34; *p* < 0.01), and participation in various types of entertainment (*p* = 0.24; *p* < 0.01). Analysis yielded a positive correlation between BAS activity and the amount of perceived benefits of cutting down sleep (*p* = 0.26; *p* < 0.01 for BAS Fun Seeking and *p* = 0.25; *p* < 0.01 for BAS Reward Responsiveness) and the magnitude of BIS activation and the number of perceived losses (*r* = 0.19; *p* < 0.01). Individuals with a higher BAS activity in the Reward Responsiveness subscale more often report choosing sleep deprivation voluntarily (*r* = 0.14; *p* < 0.5). The quality of sleep is related to BIS/BAS activation. The reasons for voluntary sleep deprivation differ depending on the level of BAS/BIS activation.

## Introduction

One of the most frequently mentioned factors that impact the way human organism functions is sleep quality (Matricciani et al., 2018). Although people differ in terms of how much sleep they need and their preferred sleeping and waking hours, everybody needs the optimal duration of sleep, uninterrupted (if possible) and occurring at similar times every night (Van Dongen et al., 2003). Meanwhile, the lifestyle of many people compels them to cut short the duration of their sleep and use that time for other purposes, whether imposed by external circumstances or by their own choice (Ahrberg et al., 2012).

The role of personality and temperamental factors in the etiology and course of illness has always attracted the attention of psychologists. Regularly repeated activities that affect health (health-related behavior) have significant consequences for physiological well-being. Inclination toward certain health-promoting and health-risk behaviors is closely related to individual personality and temperamental traits (Kane et al., 2004; Vollrath and Torgersen, 2005).

The biopsychological model of personality has been used in the past to explain why people engage in behaviors that are risky for individual health (Braddock et al., 2011), such as: abuse of alcohol (Garcia, 2010; Wardell et al., 2011) and other legal and illicit psychoactive substances (Taylor et al., 2006; Penolazzi et al., 2012) risky sexual behaviors, driving without a seatbelt, poor diet and insufficient physical activity (Schneider and Graham, 2009; Voigt et al., 2009).

The internal factor that may influence the frequency and the reasons for limiting sleep time is the activity of two affective systems: Behavioral Inhibition System (BIS) and Behavioral Activation System (BAS). The BIS is responsible for sensitivity to threatening stimuli (punishments). Its increased activity regulates aversive motivation and inhibits behavior. The BAS regulates responses to reward signals and the level of approach behavior with respect to a desired object or goal (Gray, 1987, 1990).

Voluntary sleep deprivation is another health-risk behavior the rate of which may be influenced by temperamental factors. Sleep disturbances are among the most common health problems. Poor sleep quality may relatively quickly lead to elevated stress, low mood, decreased cognitive abilities and the general quality of life (Aloba et al., 2007). Devoting the appropriate amount of time for sleep is one of healthy behaviors that have positive effects on most aspects of life (Buysse et al., 1989).

Between 15 and 35% of the general population report sleep problems (Buysse et al., 1989). Young adults in developed countries are also affected by sleep disturbances: in United States, up to 41.9% of people aged 20–39 years suffer from insomnia, and the corollary of sleep problems is lower health-related quality of life (Chen et al., 2014). Moreover, college students’ sleeping habits differ from those of their non-studying peers (Aloba et al., 2007). Students are considered a high-risk group: decreased sleep quality is associated with the lifestyle that involves striving for academic and social success while often having to undertake employment (Aloba et al., 2007; Pallos et al., 2007; Suen et al., 2008; Sing and Wong, 2010). There are high rates of activities that disrupt the regular sleep and wakefulness cycle among higher education facility students. These include late night parties, studying for exams and using computers late into the night, working the evening or night shift, and substance consumption (coffee, cigarettes, alcohol) (Pallos et al., 2005, 2007; Suen et al., 2008; Sing and Wong, 2010). Despite proven links between reduced sleep time and poorer absorption of information, many students decide to engage in other activities at the expense of sleep, particularly during finals (Thacher, 2008; Ahrberg et al., 2012).

Personality and temperamental factors influence both the quality of sleep and the consequences of voluntary sleep deprivation (Killgore et al., 2007). The resulting thoughts and emotions, as well as behaviors, can contribute to sleep difficulties (Harvey, 2002). Both state and trait personality variables are associated with a variety of deficits in cognitive performance after sleep deprivation (Carlozzi et al., 2010). Sleep deprivation is on the rise in developed countries, both due to stress-relates sleep disturbances, and intentional limiting of sleep to meet obligations or engage in leisure activities for which there is insufficient time during the day. Despite the increasing affluence of societies and development of technologies that facilitate everyday life, failure to allocate sufficient time for sleep is widespread, affecting increasingly younger individuals, including college and high school students (Talbot et al., 2010). While expectations regarding school and work performance, and tolerance for competition-related stress are mounting, sleep deprivation adversely impacts intellectual ability, especially in the presence of emotional arousal (Minkel et al., 2012).

Considering how important adequate sleep (in terms of time and quality) is for the general well-being, as well as for work and academic productivity, and given the fact that voluntary sleep deprivation is seen as a normal phenomenon accompanying active lifestyle, there is every reason to investigate the factors that determine the tendency to give up sleep. When looking for individual reasons, a good strategy seems to be to focus on basic dispositional differences. BIS and BAS, the two dimensions of temperament that, according to Gray, divide people into those oriented toward approaching a reward and those who mostly avoid aversive stimuli, provide a valid explanation of differences in the willingness to cut down sleep. Significantly reducing sleep time generates discomfort but can be beneficial in terms of having more time for achieving professional, educational, and social goals, or avoiding negative consequences of neglecting important obligations.

## Aim and Hypotheses

The aim of the study was to investigate the relationships between the BIS and BAS activity and frequency of voluntary sleep deprivation, taking into account the reasons for reducing sleep time and the overall quality of sleep among college students. Higher activity of BAS was expected to correlate with more frequent voluntary sleep deprivation associated with spending more time on work, entertainment and social life, as well as greater focus on the benefits of limiting sleep time. Slightly different relationships were predicted between individual BAS dimensions and reasons for cutting down sleep: BAS Drive activation should be responsible for reducing sleep for work-related reasons, while BAS Reward Responsiveness and Fun Seeking – for reasons involving social life and various types of entertainment. BIS activity was also expected to be associated with frequent sleep deprivation due to academic obligations, perceiving more losses resulting from reducing sleep time and overall lower sleep quality. The following hypotheses were formulated:

1. The level of activation of the Behavioral Inhibition System (BIS) will be associated with the lower quality of sleep.
2. There will be a relationship between the level of activation of the Behavioral Activation System (BAS) and the reasons for deliberate abandonment of sleep:
2A. There will be a positive correlation between the level of activation of BAS Drive and variation in the frequency of giving up sleep in order to dedicate time to study and commit to paid work.
2B. The level of BAS Reward Responsiveness will be positively correlated with the amount of time devoted to acquiring additional qualifications (outside the study program) and preparation for classes at the university at the expense of sleep.
2C. The activation level of BAS Fun Seeking dimension will be positively correlated with the tendency to give up sleep in order to participate in social life and other forms of entertainment.
3. There will be a positive relationship between the level of BIS activation and the frequency of giving up sleep in order to allocate time to assimilate the content covered by the study program.
4. The level of BAS activation will be positively correlated with the number of perceived benefits of sleep restriction to accomplish other tasks.
5. The level of BIS activation will be positively correlated with the amount of perceived losses due to limitation of sleep.
6. The level of BAS Reward Responsiveness activation will be positively correlated with the perception of more reasons to deliberately limit sleep, considered as an individual choice.

## Materials and Methods

### Participants and Procedures

Participants were 223 Polish college students, 136 female and 87 male. Mean age of participants was 21.49 years (*SD* = 2.2). The survey was conducted in group conditions after obtaining the consent of the subjects. It was voluntary and anonymous. The study group included full-time students under 35 years of age. The study trial is non-clinical and the framework of the study is exploratory.

### Instruments

The following measures were used:

BIS/BAS Scales: BIS/BAS Scales (Carver and White, 1994; Gray et al., 2016) Polish adaptation by Wytykowska (Muller and Wytykowska, 2005) measures activation of the motivational systems described in RST. The scale consists of 24 items that make up 4 scales: BIS, BAS Drive, BAS Reward Responsiveness, BAS Fun Seeking. Responses are given on a 4-point scale.

Pittsburgh Sleep Quality Index (PSQI): The PSQI is an instrument used for self-assessment of sleep quality during the past month, consisting of 24 questions (Buysse et al., 1989). The last 5 questions are addressed to the roommate of the respondent and are optional. The instrument consists of seven subscales: *subjective sleep quality, sleep latency, sleep duration, habitual sleep efficiency, sleep disturbances, use of sleeping medication, daytime dysfunction.* The final score is between 0 and 21; a score ≤ 5 indicates good quality of sleep in the subject, higher scores suggest poorer sleep quality. PSQI was used to measure sleep patterns of the last 4 weeks.

A survey of frequency and reasons for voluntary sleep deprivation: To test if sleep deprivation among participants was the result of their conscious decisions, a survey of reasons for giving up on sleep was developed for the purposes of the study. Participants chose activities in which they engaged at the expense of sleep (studying, work, extracurricular education, social life, entertainment) and reported their frequency on a 5-point Likert scale. They specified whether engaging in those activities was a matter of necessity or choice, each time providing a reason. They were also asked to select from a list of benefits and losses associated with limiting sleep time for the sake of other activities. The survey concerned the habits and life choices of the last year. Sample questions of the survey were:

1. “Have you ever taken other activities during your sleep period during the last year? If so please indicate below what kind of activities and how often they were taken at the expense of sleep.” List of possible answers: education; work; social life; additional training; entertainment, other (what?).
2. “What benefits do you see in undertaking various activities at the expense of sleep (you can choose more than one answer)?”: List of possible answers: better academic performance; higher income; more satisfying social life; more time for entertainment; the ability to reconcile several life roles; other (what?); I do not see any benefits.

### Statistical Analysis

Statistical analysis was conducted using Mann–Whitney’s *U*-test and Spearman’s rho correlation. The non-parametrical measures were chosen as a better match to the data distribution. Statistical analysis was conducted with use of IBM SPSS v. 24 statistical package.

## Results

97.3% of participants reported regularly sleeping less than their ideal sleep duration to engage in other activities. The quality of participants’ sleep was slightly below normal range (*M* = 5.5; *SD* = 2.68), especially among women (*M* = 5.89; *SD* = 2.68). Table 1 shows the results of the subjects on BIS and BAS scales.

**Table 1:** Results of the subjects tested on the BIS/BAS scale.

BIS/BAS scale	Min.	Max.	Mean	*SD*
BAS drive	4	16	10.22	2.61
BAS fun seeking	4	16	11.31	2.57
BAS reward responsiveness	8	20	16.52	2.43
BIS	9	28	21.61	4.19

Mean sleep quality among male participants was within norm (*M* = 4.9; *SD* = 2.59). There were significant differences in the quality of sleep of female participants compared to males. The Mann–Whitney’s *U*-value was *U* = 4525.5; *p* < 0.01. Mean rank of sleep quality assessment was 122.22 for women and 96.02 for men. This means that women gave lower ratings to their sleep quality. Table 2 shows the relationships between the magnitude of BIS and BAS activation and sleep quality. A significant correlation in the study group was found between the level of BIS activity and sleep quality assessment, calculated using Spearman’s *rho* (*r*_s_ = 0.26; *p* < 0.01), consistent with Hypothesis 1. Due to significant differences between women and men with respect to both correlated variables, the results are also presented separately for each gender. The correlation proved significant in the group of male participants (*r*_s_ = 0.23; *p* < 0.05). In women the result was below significance threshold (*r*_s_ = 0.17; *p* = 0.054), although the value still indicated a tendency similar to the one found in men, i.e., that higher levels of activation of BIS are associated with lower ratings of sleep quality.

**Table 2:** Dependence of activation of BIS and BAS behavioral systems and the quality of sleep.

BIS/BAS Scale	PSQI		
	All	Women	Men
BAS drive	−0.05	−0.04	0.0
BAS fun seeking	0.09	0.05	0.16
BAS reward responsiveness	0.0	−0.12	0.14
BIS	0.26^∗∗^	0.17	0.23^∗^
		*p* = 0.054	

*Probability note: ^∗^p < 0.05; ^∗∗^p < 0.01.*

Participants were asked to indicate on a five-point scale how often they engaged in particular activities instead of sleeping. Table 3 presents the relationship between BIS and BAS activation levels and frequency of performing selected activities at the expense of sleep. There was a significant correlation between the magnitude of BAS activation in the Fun Seeking scale and the frequency of reducing sleep time to engage in social activities (*r*_s_ = 0.34; *p* < 0.01) and participation in various types of entertainment (*r*_s_ = 0.24; *p* < 0.01) which confirms the Hypothesis 2 only partially. Results did not support the hypothesized (Hypothesis 2A and 2B) correlations between the activity of BAS Drive and Reward Responsiveness and reasons for voluntary sleep deprivation. Furthermore, the analysis revealed a relationship between the level of BAS Fun Seeking activity and reducing sleep time more frequently due to work (*r*_s_ = 0.18; *p* < 0.05) which was not hypothesized. The positive correlation between BIS activation and frequency of studying and preparing for academic classes at the expense of sleep was confirmed (*r*_s_ = 0.19; *p* < 0.01), consistent with Hypothesis 3.

**Table 3:** Dependence of activation levels of BIS and BAS and frequency of activity at the expense of sleep.

BIS/BAS scale	Frequency of activity at the expense of sleep				
	Education	Work	Social life	Additional training	Entertainment
BAS drive	−0.01	0.17	0.07	0.1	0.02
BAS fun seeking	−0.06	0.18^∗^	0.34^∗∗^	0.05	0.24^∗∗^
BAS reward responsiveness	−0.05	0.16	0.10	−0.14	−0.0
BIS	0.19^∗∗^	0.03	0.01	−0.05	−0.05

*Probability note: ^∗^p < 0.05; ^∗∗^ p < 0.01.*

The survey asked participants to indicate benefits and losses they associated with undertaking various activities instead of sleeping. Five potential benefits and seven potential losses were listed; participants could add one item to the list or choose *I do not see any benefits* or *I do not incur any losses*. There was a positive correlation between the number of perceived benefits and the level of BAS activation. This relationship was present across all BAS scales, which supports Hypothesis 4. Spearman’s *rho* values are presented in Table 4. The analysis of data confirmed the predicted (Hypothesis 5) relationship between the level of BIS activation and the number of perceived losses resulting from voluntary sleep deprivation. The number of perceived losses is positively correlated with BIS activation (*r*_s_ = 0.19; *p* < 0.01).

**Table 4:** Dependence of BIS and BAS activity and the number of reasons for resigning from sleep and the gains and losses observed by the subjects.

BIS/BAS scale	The number of named (due to the resignation from sleep)			
	Benefits	Losses	Necessities	Preferences
BAS drive	0.16^∗^	0.02	0.09	0.05
BAS fun seeking	0.26^∗∗^	−0.01	0.02	0.1
BAS reward responsiveness	0.25^∗∗^	0.11	−0.0	0.14^∗^
BIS	0.1	0.19^∗∗^	0.07	−0.03

*Probability note. ^∗^p < 0.05; ^∗∗^p < 0.01.*

Participants were asked if in their case voluntary sleep deprivation was a necessity or their own choice. They were also asked to describe reasons for their particular circumstances. Four situations necessitating sleep deprivation (external factors) and six sample reasons for making an independent choice were proposed. In addition, participants could add reasons not mentioned in the survey to the list. The analysis did not confirm the predicted positive relationship between BIS activation and the number of reasons not to sleep considered a necessity. Analysis yielded a positive correlation between the number of reasons for voluntary sleep deprivation and BAS activation in the Reward Responsiveness scale (*r*_s_ = 0.14; *p* < 0.05), consistent with Hypothesis 6.

## Discussion

It follows from our findings that the college students in the study group experience sleep disturbances at a level that may adversely impact their daily functioning (means PSQI score was 5.5 points). It should be noted that 97.3% of students in the study admitted to regularly cutting short their ideal sleep time to do other activities. Sleep quality affects the overall perceived quality of life (Leger et al., 2001; Ritsner et al., 2004). A valid conclusion is that the scope of sleep problems in the young adults’ population should feature prominently in any discussion on their quality of life.

There were significant differences in the quality of sleep of female participants compared to males. The trend of declining sleep quality in adolescence and early adulthood, more noticeable in women, has been observed for some time in developed countries (Brand et al., 2005). Deterioration in sleep quality is affecting increasingly younger people, especially teenage girls (Galland et al., 2017). Shorter sleep is associated with lower satisfaction with life, poorer family relations and academic performance among adolescents (Segura-Jimenes et al., 2015). The reasons for poorer sleep quality among adolescents and young adults in developing countries are complex. They include the mounting pressure on academic performance, as well as availability of psychoactive substances and ubiquitous use of new technology (Zhang et al., 2017).

Findings reported in the studies presented here confirm the relationship between BIS activation and worsening of sleep quality. Other authors have also noted the negative correlation between BIS activation and general well-being. For instance, higher BIS activation is thought to be the underlying cause of more frequent and intense experiencing of negative emotions such as sadness, anxiety, and stress (Heponiemi et al., 2003; Erdle and Rushton, 2010). Higher exposure to negative emotions in daily life is detrimental to sleep quality (Sing and Wong, 2010), while chronic sleep disturbances are one of the risk factors for the emergence of new or exacerbation of pre-existing mental disorders (Vollrath et al., 1989). Teenagers who sleep poorly demonstrate lower self-esteem, higher mental arousal and greater severity of psychosomatic symptoms than their better sleeping peers (Brand et al., 2005).

The correlation between BAS Fun Seeking activation and rates of voluntary sleep deprivation in favor of other beneficial activities (social occasions, entertainment or work), confirmed in the present study, is consistent with previous reports that BAS activation is associated with health-risk behaviors (Franken and Muris, 2006; Smiths and Boeck, 2006; Braddock et al., 2011). Our findings suggest that it would be worthwhile to extend research into the relationships between the activation of motivational systems and other healthy and unhealthy behavior in order to expand our understanding of their reasons on the most basic level and develop more effective prevention programs catering to individuals at risk.

Higher BIS scores were positively correlated with the frequency of sleep deprivation in order to study for subjects included in college syllabi. Activity of the BIS is the biological mechanism underlying avoidance behaviors in humans and animals (Corr, 2004) and its sensitivity to activation is subject to individual differences, leading to a discernible tendency toward behaviors, moods, and cognitive processing aimed, to a greater or lesser extent, at avoiding aversive stimulation (Gray and McNaughton, 2000). The question of the effects of BIS and BAS activity on professional efficacy has attracted attention of researchers, both with respect to success drive and goal accomplishment (thought to be associated with higher BAS Drive activation), and avoidance of unpleasant consequences of neglecting one’s obligations, including the resulting negative emotions. Previous studies have shown BIS and BAS (Drive and Reward Responsiveness) activation to be related with perfectionism (Chang et al., 2007). Higher BIS activity is also associated with lower commitment to work and higher work-related stress, especially when there is little sense of being in control of the effects of one’s work (Linden et al., 2007). More doubtful about the appropriateness of their actions individuals demonstrating higher BIS activity may perform worse when the reasons for the results of work are unclear (Chang et al., 2007). Our results showing the effects of increased BIS activity suggest that in a less complex situation, such as preparing for classes and exams based on well-defined requirements, higher proclivity for inhibition may lead to greater time investment, even at the expense of sleep time.

The most commonly reported reason for poor sleep quality among the citizens of developed countries is stress, especially stress generated by high demands associated with a given job position and high probability of being dismissed. Despite high risk of decreased productivity as a result of inadequate duration of sleep, performance of work-related duties and raising one’s qualifications at the expense of sleep time is a very common practice (Wells and Vaughn, 2012).

The adverse effects of sleep deprivation are not limited to cognitive function. Insufficient sleep may also lead to poorer coping with stress in situations that require mental effort (Minkel et al., 2012). Another significant aspect is the effect of cutting sleep short on psychological well-being. Shortage of sleep may increase susceptibility to anxiety and mental arousal, and reinforce a pessimistic outlook (Talbot et al., 2010).

Sleep disturbances, both in adults and in children, are currently considered one of the most serious health problems facing a large proportion of populations in highly developed countries (Gruber, 2013). Health effects experienced by individuals have adverse consequences for the functioning of whole societies across many domains. In USA the problem is losses in the economy due to decreased productivity of workers suffering from sleep disturbances or voluntarily reducing their sleep duration. Treatment of insomnia is costly for the healthcare system. Widespread sleep deprivation among employees coupled with daily commutes is blamed for the rising road accident figures (Wells and Vaughn, 2012). The few (so far) cases of fatal traffic accidents of US policemen (some of which are caused by falling asleep behind the wheel due to tiredness) are sometimes compared to death from overwork (jap. *karoshi*) which has affected mostly the Japanese corporations’ employees (Luo and Ruiz, 2012).

A lot of research today focuses on the relationship between personality traits and subjective well-being (Diener and Lucas, 1999). At the same time, authors pay attention to minor, everyday difficulties whose long-term effects on well-being and health depend on nervous system characteristics and psychological traits of the individual (Gable et al., 2000). Both the required sleep duration and its quality depend on the overall psychophysical condition of the individual, personality traits, and decisions and opinions regarding everyday requirements (Levin et al., 2002).

Prolonged sleep deprivation adversely impacts quality of life (Paiva et al., 2015), and empirical evidence shows that the number of reasons for sleep deprivation can be partially dependent on temperamental characteristics. This issue requires further research on larger and more diverse samples. Based on the currently available information, interventions aimed at improving sleep quality should not be limited to biological causes of insomnia, but include the psychological aspects of decreased sleep quality and take into account the phenomenon of voluntary sleep deprivation. Psychoeducation and health education would benefit from putting more emphasis on the interrelations between the psychophysical state of individuals and their everyday decisions concerning healthy living, including appropriate sleep duration. Evidence shows that among adolescents, increased awareness of the importance of sleep for the quality of performance and the combined effect of internal motivation and external motivators led to the increase in the amount of sleep by advancing bedtime (Cassoff et al., 2014). The reason why people reduce their sleep is often their desire for academic and professional success and their wish to use additional time for entertainment and building relationships. However, in the long run such attitudes carry the risk of failure due to deteriorating cognitive and physical functioning caused by sleep deprivation.

Our findings can serve as a point of departure for further inquiry into the causes of poor sleep quality among young adults in developed countries and research on effective strategies of improving sleep without disadvantaging other spheres of life. Research findings support the view of a strong relationship between behavior and quality of life and justify the recommendation of encouraging individual responsibility for shaping pro-health behaviors.

## Limitations and Future Research

One of the limitations of the study is the fact that mainly university students took part in it. They are relatively young people in a specific life situation. It would be advisable to conduct research on a more numerous and diverse age group of adults as well as to take into account variables related to health status and the burden of professional and family duties. Also, conducting a study on larger groups to obtain more statistically reliable results is suggested in the future.

Moreover, using the correlation to explore the linkages between variables, it is impossible to confirm cause and effect relationship between behavioral systems’ level of activation and sleep quality and reasons of sleep deprivation. In future studies it seems reasonable to construct a research plan that would allow more credible cause and effect inference. Also, using qualitative approach is a justifiable way to deepen the understanding of the nature of the problem of voluntary sleep restriction. The use of qualitative methods, such as structured interviews, is a valuable alternative to newly-constructed research tools such as surveys. Surveys allow to study a larger research group in a shorter time. However, they limit the insight into the individual experience of the subjects, which threatens to overlook the important causes of the studied phenomena.

The use of a self-constructed tool to measure the reasons for voluntary abandonment of sleep resulted from the need to have the survey consisted of direct questions regarding habits of the subjects. In further research, it seems reasonable to replace the tool containing closed questions with the form of qualitative research. More complex qualitative data will allow further exploration of the phenomenon.

In the study, we relied on the self-assessment of the subjects in determining the quality of their sleep. In further research it would be justified to extend the research methods to non-invasive methods of monitoring human rest and activity cycles like actigraphy.

## Ethics Statement

This study was carried out in accordance with the recommendations of the local Research Ethics Committee at the Faculty of Psychology, University of Warsaw. The protocol was approved by the above mentioned committee. All procedures performed in this study were in accordance with the ethical standards of the institutional research committee and with the Helsinki declaration.

## Author Contributions

All authors listed have made a substantial, direct and intellectual contribution to the work, and approved it for publication.

## Conflict of Interest Statement

The authors declare that the research was conducted in the absence of any commercial or financial relationships that could be construed as a potential conflict of interest.
